# Supplementary material for: Barriers and facilitators to integrating the friendship bench into non-communicable disease care in Malawi: A qualitative study
Source: PLOS Ment Health. 2025 Sep 8;2(9):e0000428. doi: 10.1371/journal.pmen.0000428 (PMC12798580; doi:10.1371/journal.pmen.0000428)
Supplement: Attachment — Submitted filename: Response to Reviewers (2).docx [file pmen.0000428.s003.docx]

|  | **Malawi Office**  UNC Project  Private Bag A-104  Lilongwe, Malawi  Phone: 01 755056  Fax: 01 755954  Email: [imofolo@unclilongwe.org](mailto:imofolo@unclilongwe.org) | **USA Office**  Division Of Infectious Diseases  Bioinformatics Bldg., CB# 7030  Univ. of North Carolina at Chapel Hill  Chapel Hill, NC 27599-7030  Phone: (919) 966-6324  Fax: (919) 966 8536  Email: [hoffmani@med.unc.edu](mailto:hoffmani@med.unc.edu) |
| --- | --- | --- |

**_____________________________________________________________________________________________**

6 August 2025

Lambert Zixin Li
Academic Editor
PLOS Mental Health

**PMEN-D-25-00236: Barriers and facilitators to integrating the Friendship Bench into Non-Communicable Disease care in Malawi: a qualitative study**

Dear Dr. Li,

I write to resubmit the above-named manuscript for your review and approval.

We appreciate the Reviewers’ review and comments in your letter dated June 19, 2025. We have addressed each concern as follows:

**Reviewer #1:**

**1. Barriers/facilitators are listed but not mapped rigorously to CFIR domains (e.g., "lack of supervision" should link to Inner Setting to Implementation Climate).**

The multilevel barriers and facilitators in the Results section are now mapped to specific CFIR domains and constructs. Specifically, we have added text in each section of the results to link each barrier/facilitator within the CFIR context. For example, for patient-level barriers we have included the following statement:

“Patient-level barriers to successful implementation of the FB for NCD patients with comorbid depression include a lack of clinical knowledge about mental disorders, specifically depression. This barrier is relevant to the Knowledge and Beliefs about the Intervention construct within the Characteristics of Individuals domain of the CFIR and the Patient Needs and Resources construct within the Outer Setting domain as mental health knowledge, sociocultural values and beliefs may impede patient engagement and participation in psychosocial treatment.[24, 26]”

We used the original CFIR (2009)[1] and cited the framework throughout the manuscript.

**2. Remote counseling challenges are noted (p.16) but not integrated into the analysis. Pandemic disruptions (e.g., staff shortages, resource diversion) likely amplified institutional barriers but are unexplored.**

To address this concern regarding the integration of pandemic disruptions, including remote counseling challenges, into the analysis, the remote counseling challenges were mapped to CFIR domains.

The Institutional- and Implementation-Level Barriers subsection within the Results section now details how the struggles caused by the switch to remote counseling resulted in reduced quality of care. Specific points were made in this new section detailing the impacts of the lack of materials and equipment on the implementation of the FB in the remote setting and the impact on the lack of available resources on maintaining patient contact and engagement.

Beyond switching to remote counselling and the associated challenges outlined above, no further pandemic-related disruptions were reported by the study team.

**3. Page 8. Insufficient detail on how CFIR constructs were applied. The "iterative codebook refinement" process lacks specifics (e.g., how discrepancies among coders were resolved).**

To better explain how CFIR constructs were applied to this study, we have added the following explanation in Lines 179-184: “A deductive codebook was then developed by the study team to categorize interview responses regarding barriers and facilitators according to the CFIR, organizing the identified multilevel barriers and facilitators within its framework.[24] These codes were primarily designed to assess data within the Outer Setting and Inner Setting domains of the CFIR, such as the needs of the intervention receivers, or the patients (Outer Setting), and the needs of intervention deliverers, the FB counsellors (Inner Setting).[24, 26]”

This explanation directly applies the data analysis methodology to the CFIR. We have also added more detail regarding the application of CFIR to the results section.

Additionally, the iterative codebook refinement process was described in further detail in Lines 184-190: “Each of the 4 researchers first coded 4 transcripts separately and then collaboratively reviewed the coding through a line-by-line comparison process. This process involved members of the coding team explaining their individual coding application and understanding of each code’s definition until they collectively agreed upon a standardized use of the codebook. Consequently, the codebook was updated through an iterative process throughout this double coding process. Three researchers then independently coded the remaining transcripts in Dedoose. [27] ”[2]

**4. Acceptability/feasibility are assessed anecdotally. No structured metrics (e.g., fidelity checks, attendance rates, refusal logs) are reported.**

The acceptability and feasibility of implementing the FB were assessed qualitatively through the interviews, as described. The use of structured metrics to assess fidelity was outside of the scope of this qualitative, exploratory study, but were used in the evaluation of the larger SHARP trial.[3]

**5. Prevalence estimates for depression among NCD patients rely on disparate sources (some not Malawi‐specific) and some are dated (e.g., Udedi 2014). A clearer synthesis of current Malawian data—particularly post‐COVID—would strengthen the rationale.**

Studies conducted in 2020 or more recently that are specific to Malawi have been added to more accurately depict the prevalence estimates for depression among NCD patients in Malawi. The following sentences have been added to the first paragraph of the Introduction section to include these new studies to provide a clearer synthesis of more current Malawian data: “The prevalence of depression is 2-4 times higher in those living with NCDs than those living without, presenting a major burden to low- and middle-income countries (LMICs) with limited mental healthcare infrastructure to diagnose and treat depression. [3] Though limited data exists on the prevalence of depression among people living with NCDs in Malawi, a recent study at an NCD clinic in Lilongwe found that 18% of adults with type 2 diabetes screened positive for major depression.[5] The same study found that none of these cases of major depression had been detected by NCD clinicians, [5] presenting a public health threat to those living with NCDs. The high prevalence rate of depression among NCD patients, combined with the failure of primary care clinicians to diagnose and manage comorbid depression, [2] suggests that there is an urgent need to integrate mental health treatment into NCD care.[10]”

**6. Sampling problem is hard to solve: Convenience sampling of 6 patients per clinic risks selection bias and limits transferability; there is no explanation of how saturation was assessed or reached. And gender imbalance: 10/12 patient participants are female (Results, Table 1), yet the authors note the NCD population is predominantly male. The potential impact of this skew is not discussed.**

We recognize the limitations of using a small, convenience sample. During the SHARP trial, a total of 78 clients at Bwaila Hospital and 100 at Zomba Central Hospital started the FB intervention. We then interviewed 6 clients from each site.

We have added the following text to the limitations section to better discuss the gender imbalance: “Given gendered differences in comfort around discussing mental health and seeking mental healthcare services, it is possible that our female participants had different perspectives than most males who received the intervention. However, our data does not suggest this is a large concern.”

**7. The paper acknowledges sampling and social desirability biases but fails to consider translation loss, interviewer effects, and site‐specific resource disparities (e.g., Bwaila vs. Zomba)**.

Translation loss, interviewer effects, and site-specific resource disparities between the two study sites were added to the Discussion section as study limitations. The potential impact of all of these study limitations on the results of this study were also discussed. The following was added: “Beyond social desirability bias effects, interviews may have been impacted by interviewer effects such as choices in wording and body language reactions. Prior to the implementation of the intervention, interviewers were trained to use nonjudgmental and standardized language to mitigate interviewer effects. Translation loss, such as a distortion of meaning once content was translated from Chichewa to English for the analysis of the qualitative data, may also have impacted the results of this study despite the use of professional translators and bilingual study team members. Differences in resource availability at the two sites may have also skewed the experience of FB receivers and deliverers.”

**Reviewer #2**

**MAJOR ISSUES:**
 **1. I appreciate the effort to describe the methodology in detail, including various actors involved in the intervention and how they were recruited. However, the intervention itself is still not very clear. Even if (and it is not clear yet) the intervention is described elsewhere in another paper, I recommend a section in the introduction of the paper, even if it is just a paragraph or two, that describes or summarizes the FB intervention to aid the reader.**

To better summarize the Friendship Bench intervention, the following sentences have been added and revised in the Introduction section:

“Originally developed in Zimbabwe, the Friendship Bench intervention consists of six counselling sessions focused on identifying and prioritizing a patient’s problems, developing coping strategies and achievable solutions, and encouraging the patient to take action to address these problems.[16, 17] Trained lay healthcare workers provide FB in private settings allowing patients to maintain confidentiality and talk freely with their counsellor, especially in settings like Malawi where mental health is heavily stigmatized.[18, 19] This intervention has recently been adapted for use in Malawi to improve depression symptoms in adults living with HIV.[20, 21] These studies on the use of the FB in Malawi have suggested its feasibility, acceptability, and efficacy for a variety of patient populations, making it an ideal candidate for addressing the mental healthcare gap for patients living with NCDs and mild depression in Malawi.”

**2. The results section of the paper is comprehensive, but it could do with restructuring to aid the reader's understanding. At the moment, the results are structured into patient-level factors, FB counsellor-level factors, etc., but the specific factors under each, whether barriers or facilitators, can be organized further as subsections. If possible, kindly situate the results within a conceptual or theoretical framework.**

We have taken this suggestion and re-organized the Results section. Our subsections within the Results section now include Patient-Level Barriers, Patient-Level Facilitators, FB Counsellor-Level Barriers, FB-Counsellor-Level Facilitators, Institutional- and Implementation-Level Barriers, and Institutional- and Implementation-Level Facilitators. The appropriate content was re-organized under these subsections to establish a clearer structure to enhance the section’s readability and logical flow.

Additionally, we have situated the data within each Results subsection within the original CFIR [1] domains and constructs, as the updated 2022 CFIR was not yet released at the time of data analysis for this study. We added text to each section of the results to explain where these barriers/facilitators are situated within the CFIR. The mapping of the results to the CFIR has also been highlighted in the Discussion section.

**MINOR ISSUES**
 **3. Besides using the inclusion criteria, could you kindly explain what informed the number of FB users, counsellors, and developers that were interviewed? Was it data saturation or something else?**

Regarding the number of developers, there were only two individuals that led the development of the FB intervention. This has been made clearer in the text as mentions of the developers specifically state that these participants were the two original developers of the FB.

Regarding counselors, of the five FB counselors hosted at each site, we managed to interview 4, based on the availability of those counselors. The following sentence was added to the Study Population and Recruitment section: “Of the 10 FB counsellors involved in the delivery of the intervention, 8 were able to participate in the interview based on availability.”

Regarding the patients, by rule of thumb for expectation of data saturation, we strove to include at least 10 participants from this group, also taking into account resource and time constraints. Additionally, 78 clients from Bwaila Hospital and 100 from Zomba Central Hospital started FB in the SHARP trial, and we interviewed 6 clients from each study site. The following sentence was added to the Study Population and Recruitment section: “The number of patients meets the expectations for data saturation while also taking into account resource and time constraints.”

We sincerely thank the reviewers for their valuable input, which has significantly improved the manuscript. We hope that the revised version now meets the expectations of the journal, and we remain grateful for your consideration.

Sincerely,

Katherine Waddell

UNC Project-Malawi

katygwaddell@gmail.com

**Sources:**

1.Damschroder LJ, Aron DC, Keith RE, Kirsh SR, Alexander JA, Lowery JC. Fostering implementation of health services research findings into practice: a consolidated framework for advancing implementation science. Implement Sci. 2009;4.

2.Dedoose L. Dedoose version 9.0. 85 cloud application for managing, analyzing, and presenting qualitative and mixed method research data. Los Angeles, CA: SocioCultural Research Consultants, LLC. 2021.

3.Gaynes BN, Akiba CF, Hosseinipour MC, Kulisewa K, Amberbir A, Udedi M, et al. The Sub-Saharan Africa Regional Partnership (SHARP) for Mental Health Capacity-Building Scale-Up Trial: Study Design and Protocol. Psychiatric services (Washington, DC). 2021;72(7):812-21.
